# Supplementary material for: Airborne Microplastic in the Atmospheric Deposition and How to Identify and Quantify the Threat: Semi-Quantitative Approach Based on Kraków Case Study
Source: Int J Environ Res Public Health. 2022 Sep 27;19(19):12252. doi: 10.3390/ijerph191912252 (PMC9564561; doi:10.3390/ijerph191912252)
Supplement: Supplementary file 1 [file ijerph-19-12252-s001.zip › ijerph-1860508-supplementary.pdf]

Project: Michalik19

BK 1

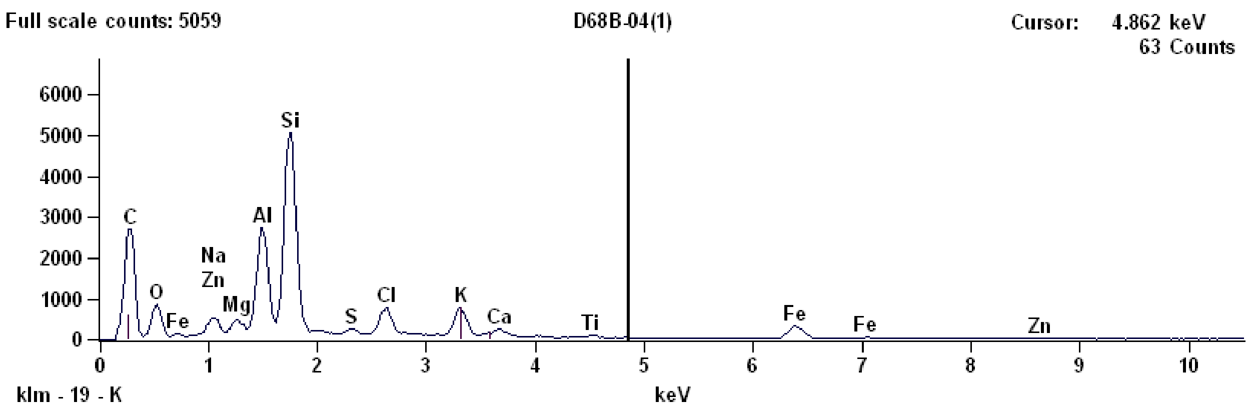

Live Time: 100.0 sec.

Fri Jan 10 14:10:34 2020
Filter Fit Chi Squared:3.711
Errors: +/- 1 Sigma
Correction Method: Proza (Phi-Rho-Z)
Acc.Voltage: 20.0 kV Take Off Angle: 28.5 deg.

Quantitative Results for: D68B-04(1)

Element Line	Net Counts	Net Counts Error		Weight %	Weight % Error	Atom %	Atom % Error	Formula	Compnd %	# Cations
O K	6572	+/-	129	35.35	+/- 0.69	50.99	+/- 1.00	O	35.35	---
Na K	2620	+/-	133	2.90	+/- 0.15	2.91	+/- 0.15	Na	2.90	1.369
Mg K	1854	+/-	157	1.30	+/- 0.11	1.23	+/- 0.10	Mg	1.30	0.580
Al K	25455	+/-	284	12.91	+/- 0.14	11.04	+/- 0.12	Al	12.91	5.198
Si K	55609	+/-	387	28.59	+/- 0.20	23.49	+/- 0.16	Si	28.59	11.057
S K	1349	+/-	89	0.77	+/- 0.05	0.55	+/- 0.04	S	0.77	0.261
Cl K	8324	+/-	195	4.89	+/- 0.11	3.18	+/- 0.07	Cl	4.89	1.498
K K	8805	+/-	207	5.06	+/- 0.12	2.99	+/- 0.07	K	5.06	1.405
Ca K	2101	+/-	163	1.27	+/- 0.10	0.73	+/- 0.06	Ca	1.27	0.344
Ti K	399	+/-	58	0.31	+/- 0.05	0.15	+/- 0.02	Ti	0.31	0.071
Fe K	5175	+/-	115	6.25	+/- 0.14	2.58	+/- 0.06	Fe	6.25	1.215
Zn K	173	+/-	58	0.42	+/- 0.14	0.15	+/- 0.05	Zn	0.42	0.070
Total				100.00		100.00			100.00	23.068

BK 2

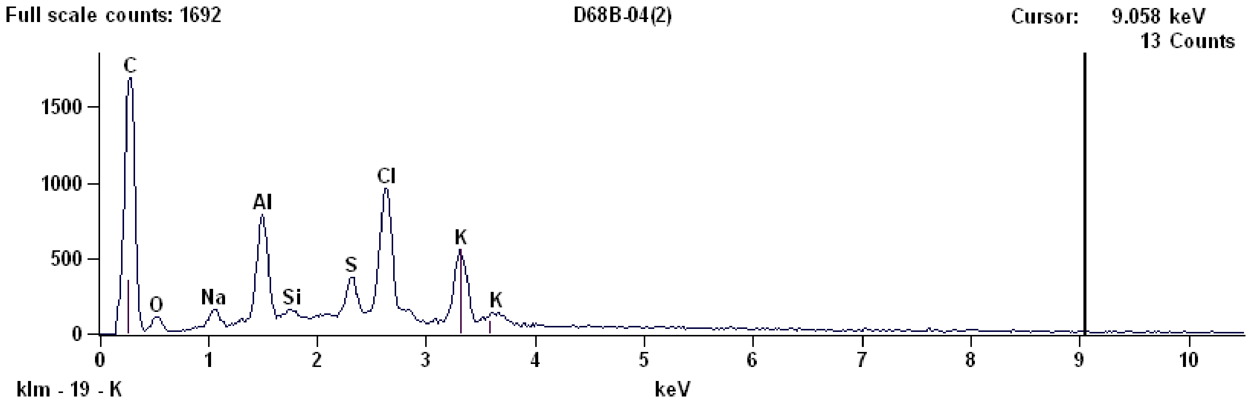

Live Time: 100.0 sec.

Fri Jan 10 14:13:24 2020
Filter Fit Chi Squared:7.640
Errors: +/- 1 Sigma
Correction Method: Proza (Phi-Rho-Z)
Acc.Voltage: 20.0 kV Take Off Angle: 28.5 deg.

Quantitative Results for: D68B-04(2)

Element Line	Net Counts	Net Counts Error		Weight %	Weight % Error	Atom %	Atom % Error	Formula	Compnd %	# Cations
O K	708	+/-	47	28.25	+/- 1.88	44.41	+/- 2.95	O	28.25	---
Na K	942	+/-	59	4.50	+/- 0.28	4.93	+/- 0.31	Na	4.50	2.663
Al K	7221	+/-	119	16.07	+/- 0.26	14.98	+/- 0.25	Al	16.07	8.095
Si K	416	+/-	59	0.98	+/- 0.14	0.88	+/- 0.12	Si	0.98	0.475
S K	2900	+/-	90	5.68	+/- 0.18	4.46	+/- 0.14	S	5.68	2.409
Cl K	11358	+/-	204	25.95	+/- 0.47	18.41	+/- 0.33	Cl	25.95	9.950
K K	6769	+/-	164	18.56	+/- 0.45	11.94	+/- 0.29	K	18.56	6.451
Total				100.00		100.00			100.00	30.043

BK 3

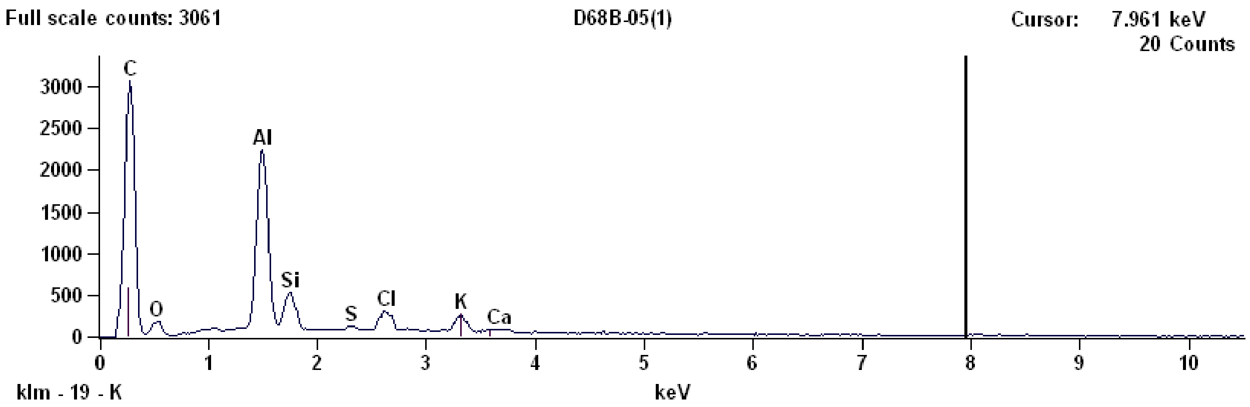

Live Time: 100.0 sec.

Fri Jan 10 14:19:17 2020
Filter Fit Chi Squared:7.245
Errors: +/- 1 Sigma
Correction Method: Proza (Phi-Rho-Z)
Acc.Voltage: 20.0 kV Take Off Angle: 28.4 deg.

Quantitative Results for: D68B-05(1)

Element Line	Net Counts	Net Counts Error	Weight %	Weight % Error	Atom %	Atom % Error	Formula	Compnd %	# Cations
O K	1256	+/- 59	31.19	+/- 1.46	45.21	+/- 2.12	O	31.19	---
Al K	22746	+/- 211	38.77	+/- 0.36	33.33	+/- 0.31	Al	38.77	17.693
Si K	4827	+/- 146	12.94	+/- 0.39	10.69	+/- 0.32	Si	12.94	5.673
S K	605	+/- 63	1.40	+/- 0.15	1.01	+/- 0.11	S	1.40	0.537
Cl K	3234	+/- 134	7.75	+/- 0.32	5.07	+/- 0.21	Cl	7.75	2.692
K K	2602	+/- 133	6.22	+/- 0.32	3.69	+/- 0.19	K	6.22	1.957
Ca K	691	+/- 114	1.74	+/- 0.29	1.01	+/- 0.17	Ca	1.74	0.534
Total			100.00		100.00			100.00	29.086

BK 4

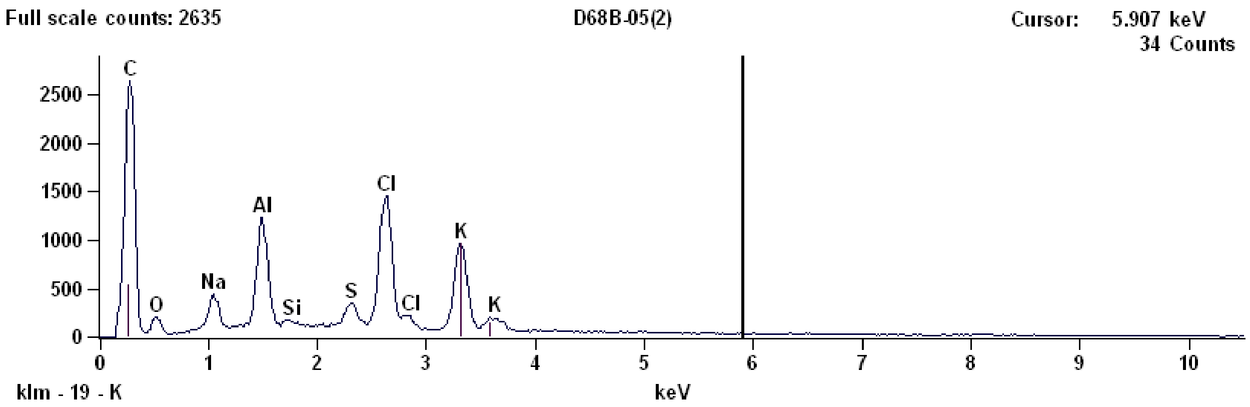

Live Time: 100.0 sec.

Fri Jan 10 14:21:49 2020
Filter Fit Chi Squared:9.692
Errors: +/- 1 Sigma
Correction Method: Proza (Phi-Rho-Z)
Acc.Voltage: 20.0 kV Take Off Angle: 28.4 deg.

Quantitative Results for: D68B-05(2)

Element Line	Net Counts	Net Counts Error		Weight %	Weight % Error	Atom %	Atom % Error	Formula	Compnd %	# Cations
O K	1195	+/-	61	27.74	+/- 1.42	43.27	+/- 2.21	O	27.74	---
Na K	3059	+/-	87	8.57	+/- 0.24	9.31	+/- 0.26	Na	8.57	5.162
Al K	11453	+/-	147	16.03	+/- 0.21	14.83	+/- 0.19	Al	16.03	8.225
Si K	384	+/-	64	0.56	+/- 0.09	0.50	+/- 0.08	Si	0.56	0.277
S K	2741	+/-	166	3.25	+/- 0.20	2.53	+/- 0.15	S	3.25	1.404
Cl K	17907	+/-	241	24.01	+/- 0.32	16.90	+/- 0.23	Cl	24.01	9.375
K K	12380	+/-	202	19.83	+/- 0.32	12.66	+/- 0.21	K	19.83	7.021
Total				100.00		100.00			100.00	31.464

Project: Michalik19

BK 5

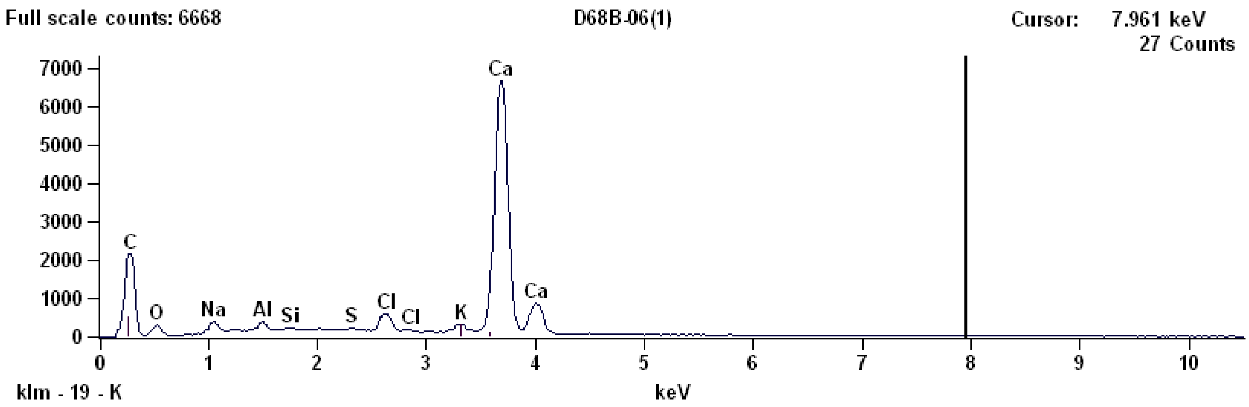

Live Time: 100.0 sec.

Fri Jan 10 14:33:33 2020
Filter Fit Chi Squared:6.106
Errors: +/- 1 Sigma
Correction Method: Proza (Phi-Rho-Z)
Acc.Voltage: 20.0 kV Take Off Angle: 28.7 deg.

Quantitative Results for: D68B-06(1)

Element Line	*Net Counts*	*Net Counts Error*	*Weight %*	*Weight % Error*	*Atom %*	*Atom % Error*	*Formula*	*Compnd %*	*# Cations*
O K	2218	+/- 72	32.31	+/- 1.05	52.84	+/- 1.72	O	32.31	---
Na K	2576	+/- 88	4.26	+/- 0.15	4.85	+/- 0.17	Na	4.26	2.202
Al K	1876	+/- 111	1.28	+/- 0.08	1.24	+/- 0.07	Al	1.28	0.564
Si K	718	+/- 74	0.39	+/- 0.04	0.37	+/- 0.04	Si	0.39	0.166
S K	1154	+/- 163	0.53	+/- 0.08	0.44	+/- 0.06	S	0.53	0.198
Cl K	6162	+/- 186	3.11	+/- 0.09	2.30	+/- 0.07	Cl	3.11	1.043
K K	2640	+/- 93	1.34	+/- 0.05	0.90	+/- 0.03	K	1.34	0.408
Ca K	94347	+/- 560	56.78	+/- 0.34	37.07	+/- 0.22	Ca	56.78	16.838
Total			100.00		100.00			100.00	21.419

BK 6

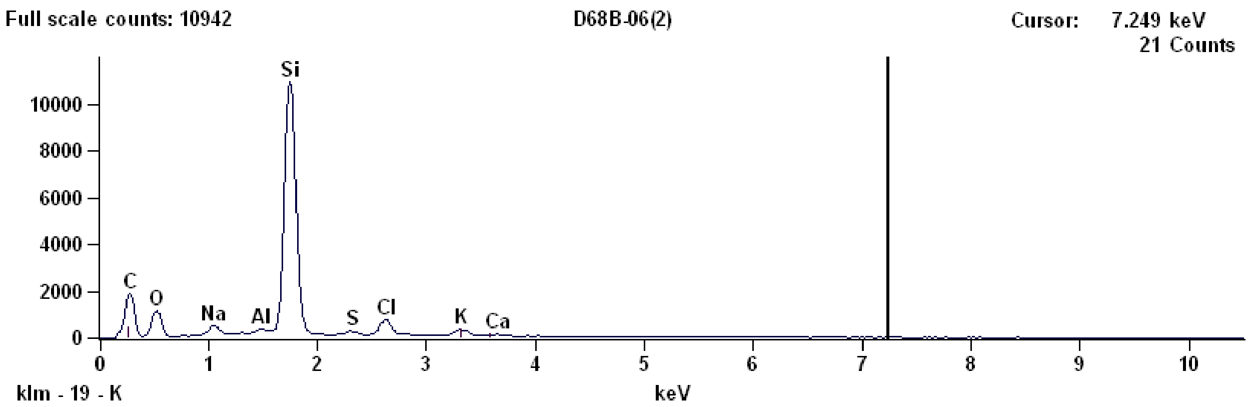

Live Time: 100.0 sec.

Fri Jan 10 14:36:10 2020
Filter Fit   Chi Squared:6.462
Errors: +/- 1 Sigma
Correction Method: Proza (Phi-Rho-Z)
Acc.Voltage: 20.0 kV Take Off Angle: 28.7 deg.

Quantitative Results for: D68B-06(2)

Element Line	*Net Counts*	*Net Counts Error*	*Weight %*	*Weight % Error*	*Atom %*	*Atom % Error*	*Formula*	*Compnd %*	*# Cations*
O K	9518	+/- 127	43.27	+/- 0.58	57.69	+/- 0.77	O	43.27	---
Na K	3967	+/- 103	3.62	+/- 0.09	3.36	+/- 0.09	Na	3.62	1.398
Al K	1163	+/- 92	0.49	+/- 0.04	0.39	+/- 0.03	Al	0.49	0.162
Si K	121358	+/- 553	44.27	+/- 0.20	33.63	+/- 0.15	Si	44.27	13.989
S K	1860	+/- 155	1.00	+/- 0.08	0.67	+/- 0.06	S	1.00	0.277
Cl K	8532	+/- 193	4.72	+/- 0.11	2.84	+/- 0.06	Cl	4.72	1.183
K K	4105	+/- 167	2.21	+/- 0.09	1.20	+/- 0.05	K	2.21	0.501
Ca K	740	+/- 140	0.41	+/- 0.08	0.22	+/- 0.04	Ca	0.41	0.091
Total			100.00		100.00			100.00	17.601

BK 7

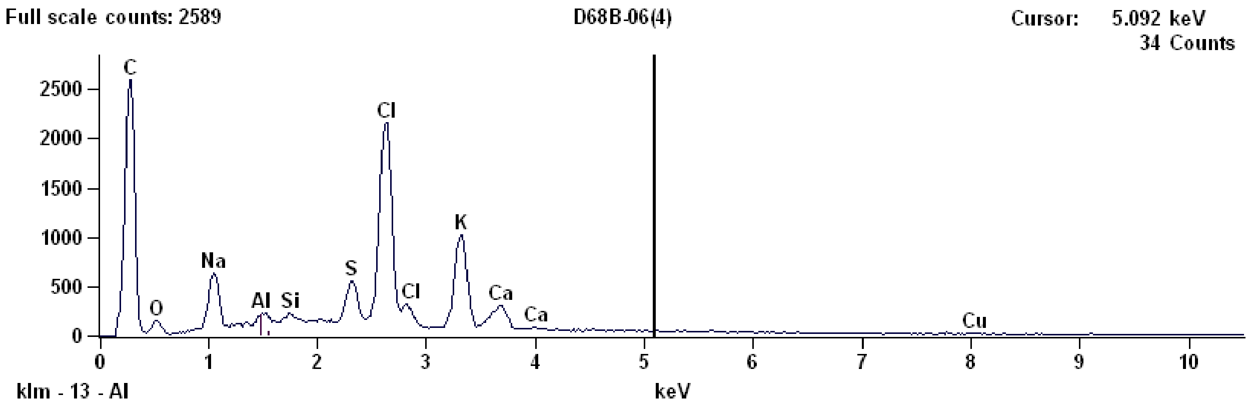

Live Time: 100.0 sec.

Fri Jan 10 14:40:04 2020
Filter Fit Chi Squared:3.393
Errors: +/- 1 Sigma
Correction Method: Proza (Phi-Rho-Z)
Acc.Voltage: 20.0 kV Take Off Angle: 28.7 deg.

Quantitative Results for: D68B-06(4)

Element Line	*Net Counts*	*Net Counts Error*		*Weight %*	*Weight % Error*	*Atom %*	*Atom % Error*	*Formula*	*Compnd %*	*# Cations*
O K	898	+/-	54	22.11	+/- 1.33	36.76	+/- 2.21	O	22.11	---
Na K	5401	+/-	104	14.45	+/- 0.28	16.72	+/- 0.32	Na	14.45	10.916
Al K	833	+/-	59	1.20	+/- 0.08	1.18	+/- 0.08	Al	1.20	0.770
Si K	944	+/-	112	1.09	+/- 0.13	1.04	+/- 0.12	Si	1.09	0.676
S K	5493	+/-	193	5.51	+/- 0.19	4.57	+/- 0.16	S	5.51	2.982
Cl K	26348	+/-	288	31.44	+/- 0.34	23.59	+/- 0.26	Cl	31.44	15.400
K K	12283	+/-	211	18.54	+/- 0.32	12.61	+/- 0.22	K	18.54	8.232
Ca K	2821	+/-	84	4.80	+/- 0.14	3.18	+/- 0.09	Ca	4.80	2.078
Cu K	171	+/-	50	0.87	+/- 0.25	0.36	+/- 0.11	Cu	0.87	0.237
Total				100.00		100.00			100.00	41.290

BK 8

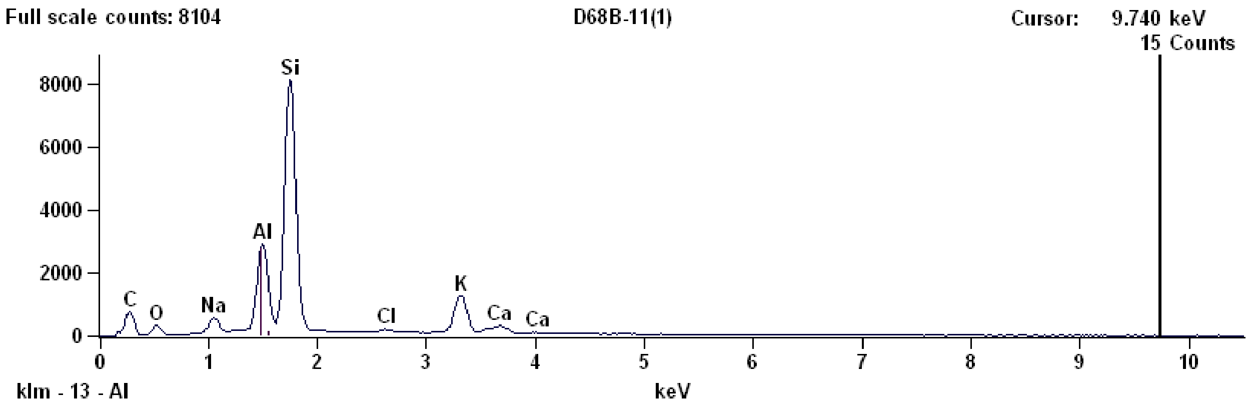

Live Time: 100.0 sec.

Fri Jan 10 14:57:00 2020
Filter Fit Chi Squared:4.049
Errors: +/- 1 Sigma
Correction Method: Proza (Phi-Rho-Z)
Acc.Voltage: 20.0 kV Take Off Angle: 30.4 deg.

Quantitative Results for: D68B-11(1)

Element Line	*Net Counts*	*Net Counts Error*	*Weight %*	*Weight % Error*	*Atom %*	*Atom % Error*	*Formula*	*Compnd %*	*# Cations*
O K	2475	+/- 69	20.29	+/- 0.57	31.57	+/- 0.88	O	20.29	---
Na K	4513	+/- 97	4.27	+/- 0.09	4.62	+/- 0.10	Na	4.27	3.514
Al K	27790	+/- 274	13.79	+/- 0.14	12.72	+/- 0.13	Al	13.79	9.667
Si K	87334	+/- 480	47.35	+/- 0.26	41.97	+/- 0.23	Si	47.35	31.902
Cl K	1088	+/- 131	0.82	+/- 0.10	0.57	+/- 0.07	Cl	0.82	0.435
K K	15736	+/- 249	11.05	+/- 0.17	7.03	+/- 0.11	K	11.05	5.347
Ca K	3196	+/- 184	2.43	+/- 0.14	1.51	+/- 0.09	Ca	2.43	1.148
Total			100.00		100.00			100.00	52.014

BK 9

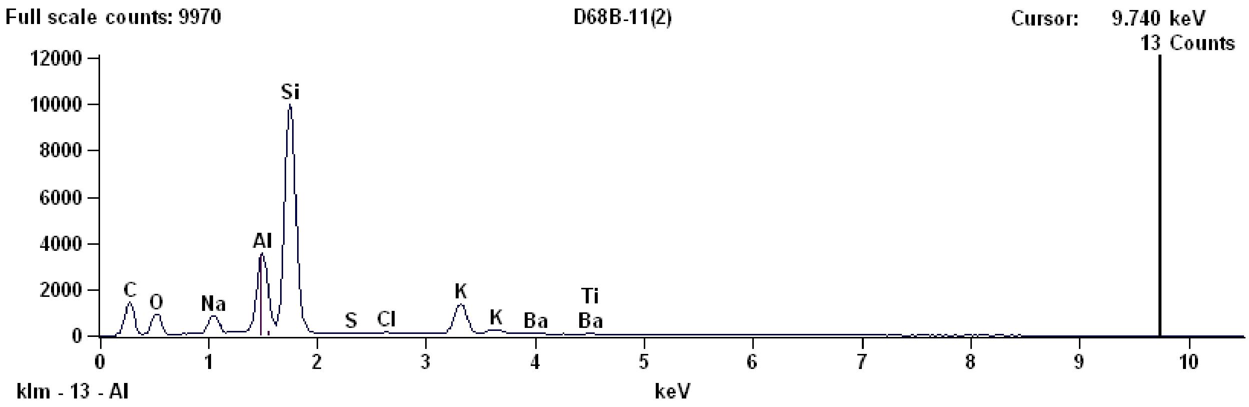

Live Time: 100.0 sec.

Fri Jan 10 14:59:40 2020
Filter Fit Chi Squared:8.519
Errors: +/- 1 Sigma
Correction Method: Proza (Phi-Rho-Z)
Acc.Voltage: 20.0 kV Take Off Angle: 30.4 deg.

Quantitative Results for: D68B-11(2)

Element Line	*Net Counts*	*Net Counts Error*	*Weight %*	*Weight % Error*	*Atom %*	*Atom % Error*	*Formula*	*Compnd %*	*# Cations*
O K	7832	+/- 115	33.79	+/- 0.50	47.66	+/- 0.70	O	33.79	---
Na K	7237	+/- 121	5.13	+/- 0.09	5.04	+/- 0.08	Na	5.13	2.536
Al K	33453	+/- 307	12.02	+/- 0.11	10.05	+/- 0.09	Al	12.02	5.064
Si K	106382	+/- 548	39.83	+/- 0.21	32.00	+/- 0.16	Si	39.83	16.117
S K	512	+/- 74	0.25	+/- 0.04	0.17	+/- 0.03	S	0.25	0.087
Cl K	636	+/- 71	0.31	+/- 0.03	0.20	+/- 0.02	Cl	0.31	0.100
K K	17826	+/- 243	8.26	+/- 0.11	4.77	+/- 0.06	K	8.26	2.400
Ti K	211	+/- 68	0.14	+/- 0.04	0.06	+/- 0.02	Ti	0.14	0.032
Ba L	287	+/- 114	0.28	+/- 0.11	0.05	+/- 0.02	Ba	0.28	0.023
Total			100.00		100.00			100.00	26.358

BK 10

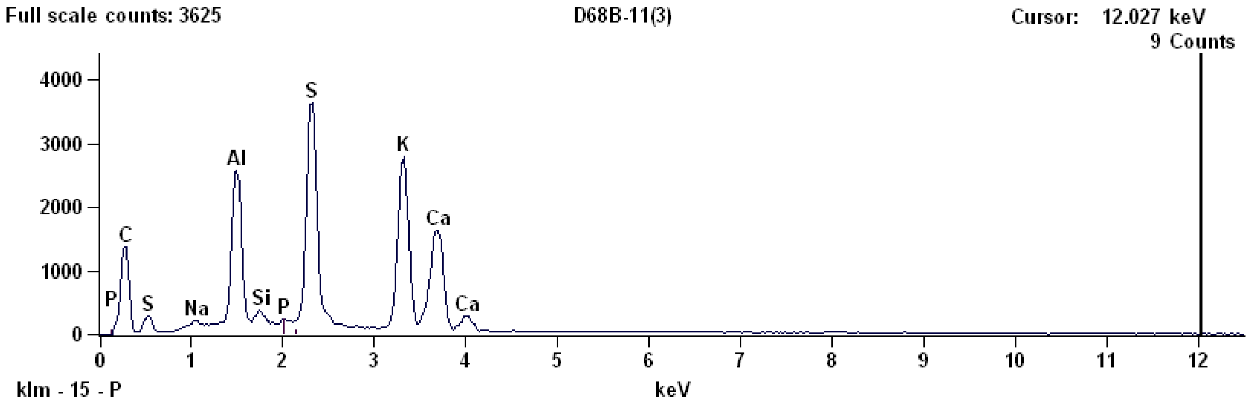

Live Time: 100.0 sec.

Fri Jan 10 15:02:29 2020
Filter Fit Chi Squared:34.329
Errors: +/- 1 Sigma
Correction Method: Proza (Phi-Rho-Z)
Acc.Voltage: 20.0 kV Take Off Angle: 30.4 deg.

Quantitative Results for: D68B-11(3)

Element Line	Net Counts	Net Counts Error	Weight %	Weight % Error	Atom %	Atom % Error	Formula	Compnd %	# Cations
Na K	873	+/- 81	1.24	+/- 0.11	1.83	+/- 0.17	Na	1.24	---
Al K	25208	+/- 227	16.39	+/- 0.15	20.69	+/- 0.19	Al	16.39	---
Si K	1919	+/- 155	1.34	+/- 0.11	1.63	+/- 0.13	Si	1.34	---
P K	345	+/- 91	0.24	+/- 0.06	0.26	+/- 0.07	P	0.24	---
S K	48030	+/- 358	29.51	+/- 0.22	31.36	+/- 0.23	S	29.51	---
K K	35629	+/- 359	29.20	+/- 0.29	25.45	+/- 0.26	K	29.20	---
Ca K	22145	+/- 311	22.08	+/- 0.31	18.77	+/- 0.26	Ca	22.08	---
Total			100.00		100.00			100.00	0.000

BK 11

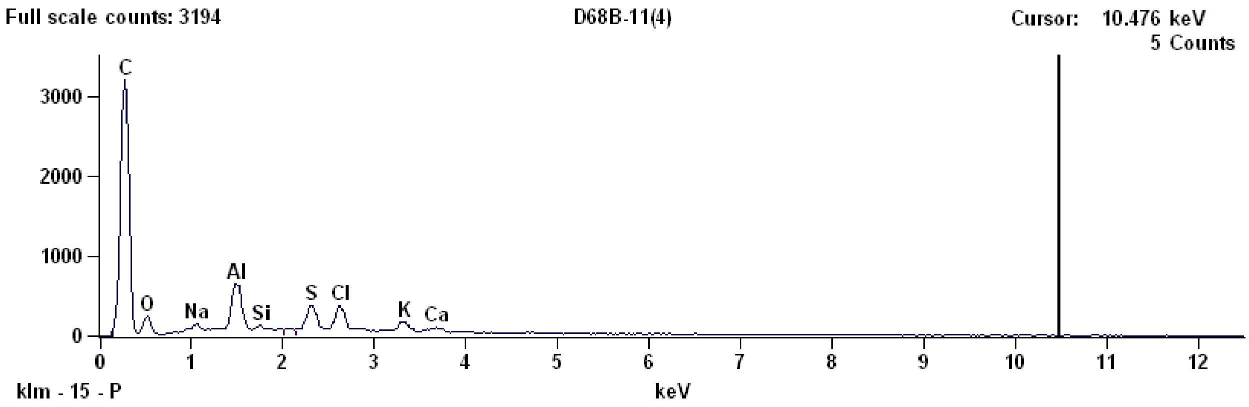

Live Time: 100.0 sec.

Fri Jan 10 15:05:06 2020
Filter Fit Chi Squared:5.325
Errors: +/- 1 Sigma
Correction Method: Proza (Phi-Rho-Z)
Acc.Voltage: 20.0 kV Take Off Angle: 30.4 deg.

Quantitative Results for: D68B-11(4)

Element Line	Net Counts	Net Counts Error		Weight %	Weight % Error	Atom %	Atom % Error	Formula	Compnd %	# Cations
O K	1644	+/-	62	47.76	+/- 1.80	63.50	+/- 2.39	O	47.76	---
Na K	804	+/-	61	4.76	+/- 0.36	4.41	+/- 0.33	Na	4.76	1.665
Al K	6123	+/-	121	16.83	+/- 0.33	13.27	+/- 0.26	Al	16.83	5.016
Si K	514	+/-	97	1.50	+/- 0.28	1.14	+/- 0.21	Si	1.50	0.429
S K	4176	+/-	144	10.26	+/- 0.35	6.81	+/- 0.23	S	10.26	2.572
Cl K	3802	+/-	143	11.23	+/- 0.42	6.74	+/- 0.25	Cl	11.23	2.546
K K	1726	+/-	115	5.41	+/- 0.36	2.95	+/- 0.20	K	5.41	1.113
Ca K	677	+/-	53	2.25	+/- 0.18	1.19	+/- 0.09	Ca	2.25	0.451
Total				100.00		100.00			100.00	13.793

BK 12

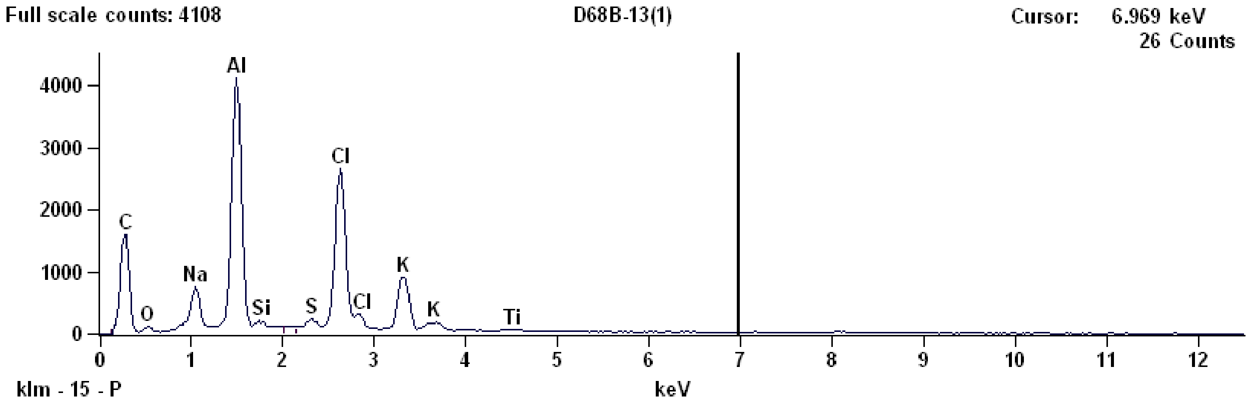

Live Time: 100.0 sec.

Fri Jan 10 15:13:13 2020
Filter Fit Chi Squared:12.028
Errors: +/- 1 Sigma
Correction Method: Proza (Phi-Rho-Z)
Acc.Voltage: 20.0 kV Take Off Angle: 30.4 deg.

Quantitative Results for: D68B-13(1)

Element Line	Net Counts	Net Counts Error	Weight %	Weight % Error	Atom %	Atom % Error	Formula	Compnd %	# Cations
O K	593	+/- 53	9.48	+/- 0.85	16.70	+/- 1.49	O	9.48	---
Na K	6017	+/- 111	8.64	+/- 0.16	10.60	+/- 0.20	Na	8.64	15.224
Al K	41754	+/- 270	33.97	+/- 0.22	35.51	+/- 0.23	Al	33.97	51.018
Si K	632	+/- 71	0.71	+/- 0.08	0.72	+/- 0.08	Si	0.71	1.029
S K	1298	+/- 83	1.14	+/- 0.07	1.00	+/- 0.06	S	1.14	1.436
Cl K	32760	+/- 299	31.17	+/- 0.28	24.79	+/- 0.23	Cl	31.17	35.620
K K	12627	+/- 198	14.46	+/- 0.23	10.43	+/- 0.16	K	14.46	14.983
Ti K	288	+/- 49	0.44	+/- 0.08	0.26	+/- 0.04	Ti	0.44	0.373
Total			100.00		100.00			100.00	119.683

BK 13

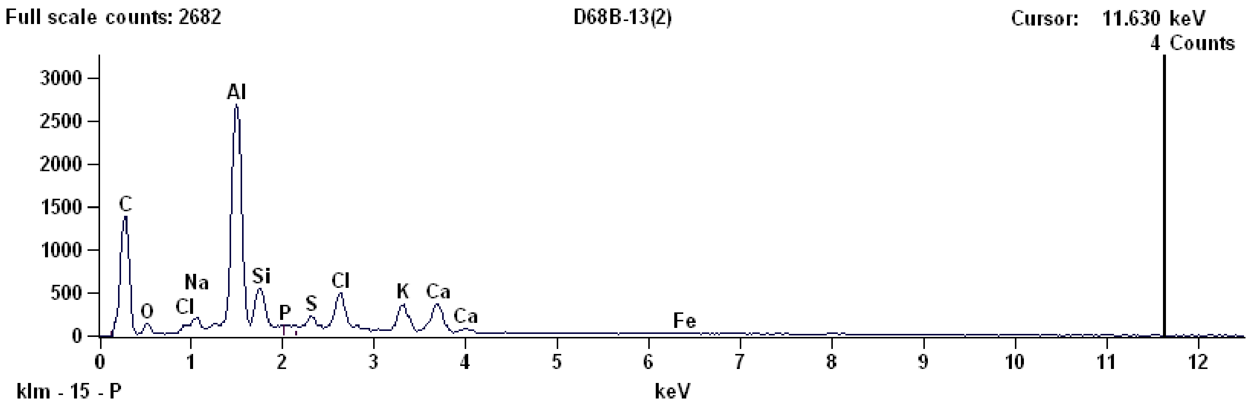

Live Time: 100.0 sec.

Fri Jan 10 15:15:49 2020
Filter Fit Chi Squared:4.299
Errors: +/- 1 Sigma
Correction Method: Proza (Phi-Rho-Z)
Acc.Voltage: 20.0 kV Take Off Angle: 30.4 deg.

Quantitative Results for: D68B-13(2)

Element Line	Net Counts	Net Counts Error		Weight %	Weight % Error	Atom %	Atom % Error	Formula	Compnd %	# Cations
O K	912	+/-	57	20.94	+/- 1.31	33.30	+/- 2.08	O	20.94	---
Na K	949	+/-	73	2.62	+/- 0.20	2.90	+/- 0.22	Na	2.62	2.090
Al K	27275	+/-	230	37.07	+/- 0.31	34.95	+/- 0.29	Al	37.07	25.192
Si K	4982	+/-	154	9.96	+/- 0.31	9.02	+/- 0.28	Si	9.96	6.502
P K	355	+/-	65	0.73	+/- 0.13	0.60	+/- 0.11	P	0.73	0.434
S K	1404	+/-	74	2.39	+/- 0.13	1.90	+/- 0.10	S	2.39	1.370
Cl K	5260	+/-	154	9.50	+/- 0.28	6.82	+/- 0.20	Cl	9.50	4.913
K K	4077	+/-	152	7.47	+/- 0.28	4.86	+/- 0.18	K	7.47	3.501
Ca K	3995	+/-	153	7.82	+/- 0.30	4.96	+/- 0.19	Ca	7.82	3.577
Fe K	386	+/-	52	1.50	+/- 0.20	0.68	+/- 0.09	Fe	1.50	0.492
Total				100.00		100.00			100.00	48.071

BK 14

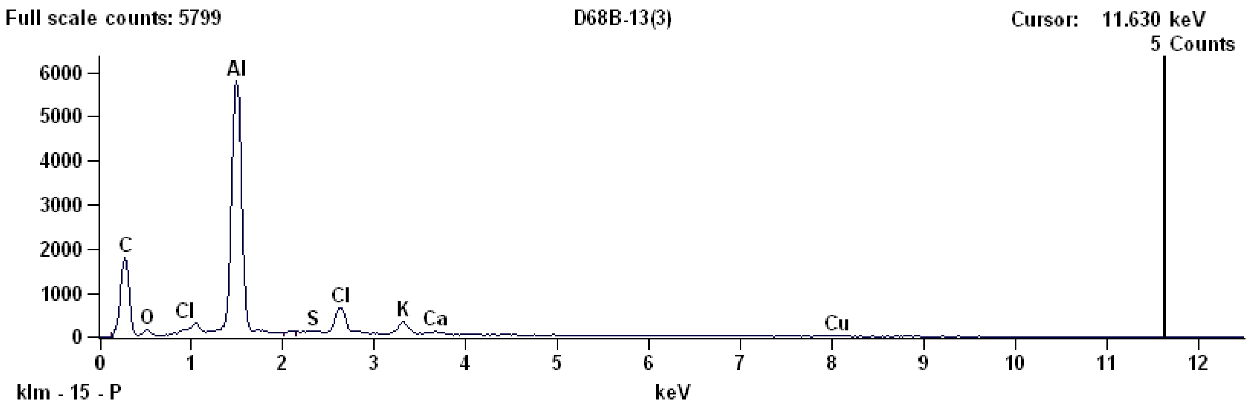

Live Time: 100.0 sec.

Fri Jan 10 15:18:16 2020
Filter Fit Chi Squared:11.609
Errors: +/- 1 Sigma
Correction Method: Proza (Phi-Rho-Z)
Acc.Voltage: 20.0 kV Take Off Angle: 30.4 deg.

Quantitative Results for: D68B-13(3)

Element Line	Net Counts	Net Counts Error		Weight %	Weight % Error	Atom %	Atom % Error	Formula	Compnd %	# Cations
O K	800	+/-	55	14.08	+/- 0.97	23.05	+/- 1.58	O	14.08	---
Al K	60444	+/-	336	63.14	+/- 0.35	61.30	+/- 0.34	Al	63.14	63.832
S K	431	+/-	69	0.67	+/- 0.11	0.55	+/- 0.09	S	0.67	0.574
Cl K	8067	+/-	170	12.99	+/- 0.27	9.60	+/- 0.20	Cl	12.99	9.994
K K	3496	+/-	145	5.75	+/- 0.24	3.85	+/- 0.16	K	5.75	4.014
Ca K	639	+/-	117	1.10	+/- 0.20	0.72	+/- 0.13	Ca	1.10	0.746
Cu K	415	+/-	52	2.27	+/- 0.28	0.93	+/- 0.12	Cu	2.27	0.973
Total				100.00		100.00			100.00	80.132

BK 15

Project: Michalik19

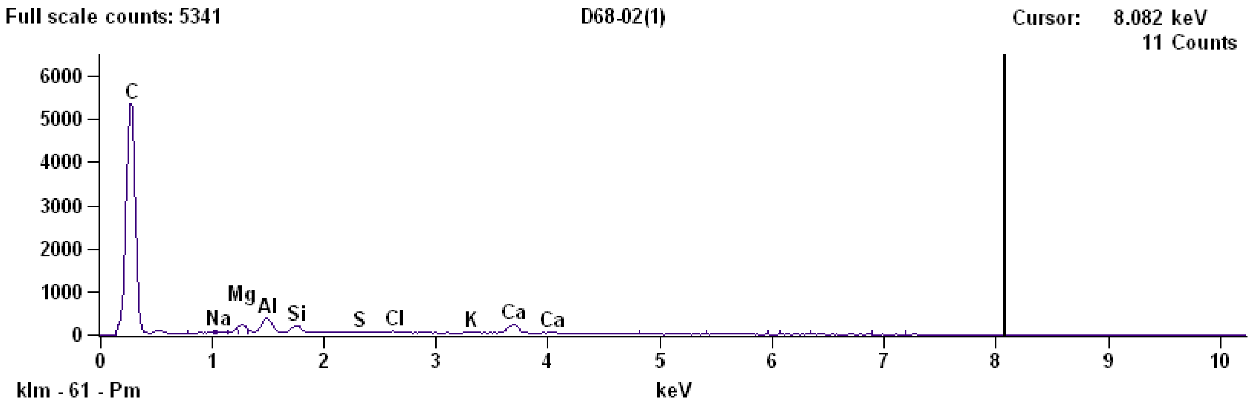

Live Time: 100.0 sec.

Fri Jan 24 12:37:36 2020
Filter Fit Chi Squared:8.141
Errors: +/- 1 Sigma
Correction Method: Proza (Phi-Rho-Z)
Acc.Voltage: 20.0 kV Take Off Angle: 29.8 deg.

Quantitative Results for: D68-02(1)

Element Line	*Net Counts*	*Net Counts Error*		*Weight %*	*Weight % Error*	*Atom %*	*Atom % Error*	*Formula*	*Compnd %*	*# Cations*
Na K	403	+/-	59	5.30	+/- 0.78	6.91	+/- 1.01	Na	5.30	---
Mg K	1412	+/-	88	13.38	+/- 0.83	16.51	+/- 1.03	Mg	13.38	---
Al K	3005	+/-	113	27.33	+/- 1.03	30.36	+/- 1.14	Al	27.33	---
Si K	1355	+/-	97	14.60	+/- 1.04	15.58	+/- 1.12	Si	14.60	---
S K	336	+/-	49	3.18	+/- 0.46	2.97	+/- 0.43	S	3.18	---
Cl K	521	+/-	46	5.18	+/- 0.46	4.38	+/- 0.39	Cl	5.18	---
K K	383	+/-	42	3.65	+/- 0.40	2.80	+/- 0.31	K	3.65	---
Ca K	2691	+/-	109	27.39	+/- 1.11	20.49	+/- 0.83	Ca	27.39	---
Total				100.00		100.00			100.00	0.000

BK 16

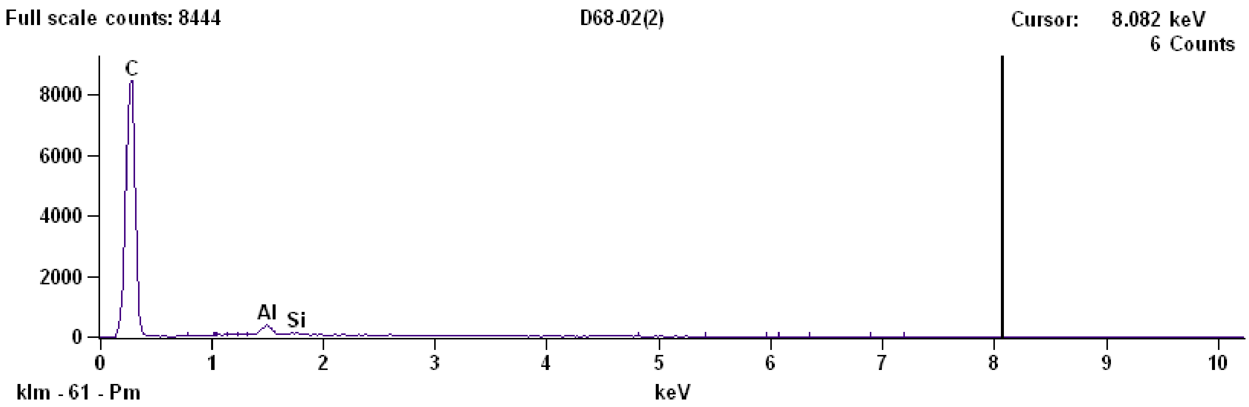

Live Time: 100.0 sec.

Fri Jan 24 12:40:39 2020
Filter Fit Chi Squared:65.140
Errors: +/- 1 Sigma
Correction Method: Proza (Phi-Rho-Z)
Acc.Voltage: 20.0 kV Take Off Angle: 29.8 deg.

Quantitative Results for: D68-02(2)

Element Line	Net Counts	Net Counts Error		Weight %	Weight % Error	Atom %	Atom % Error	Formula	Compnd %	# Cations
Al K	2967	+/-	99	68.93	+/- 2.30	69.78	+/- 2.33	Al	68.93	---
Si K	515	+/-	90	31.07	+/- 5.43	30.22	+/- 5.28	Si	31.07	---
Total				100.00		100.00			100.00	0.000

OR 1

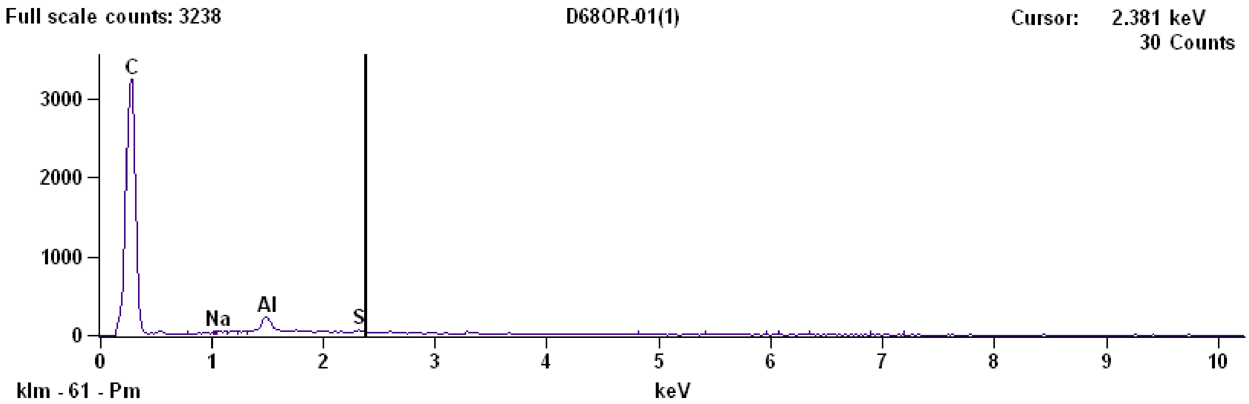

Live Time: 100.0 sec.

Fri Jan 24 12:51:26 2020
Filter Fit Chi Squared:4.781
Errors: +/- 1 Sigma
Correction Method: Proza (Phi-Rho-Z)
Acc.Voltage: 20.0 kV Take Off Angle: 29.7 deg.

Quantitative Results for: D68OR-01(1)

Element Line	Net Counts	Net Counts Error		Weight %	Weight % Error	Atom %	Atom % Error	Formula	Compnd %	# Cations
Na K	86	+/-	26	5.84	+/- 1.77	6.98	+/- 2.11	Na	5.84	---
Al K	1672	+/-	69	76.50	+/- 3.16	77.89	+/- 3.21	Al	76.50	---
S K	216	+/-	39	17.66	+/- 3.19	15.13	+/- 2.73	S	17.66	---
Total				100.00		100.00			100.00	0.000

OR 2

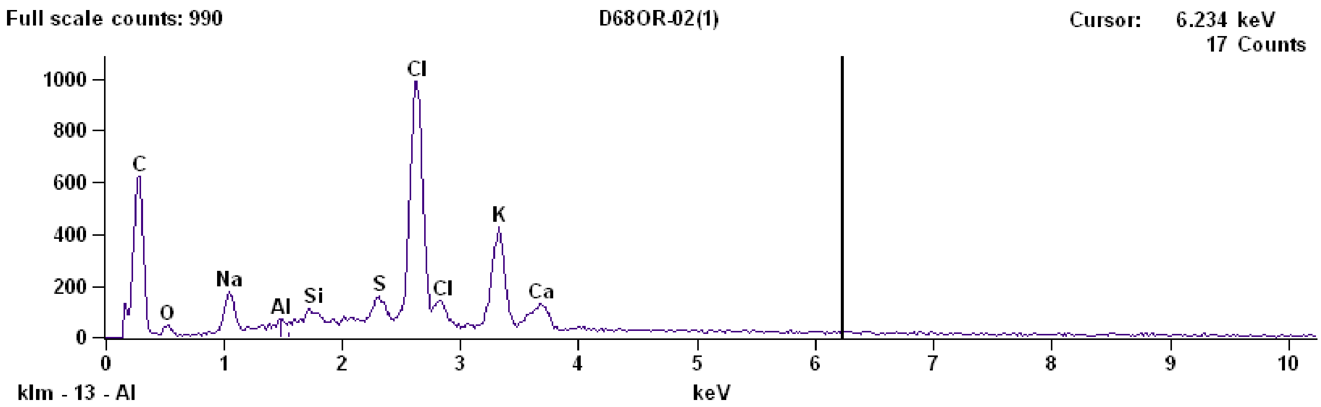

Live Time: 100.0 sec.

Fri Jan 24 13:02:27 2020
Filter Fit Chi Squared:3.577
Errors: +/- 1 Sigma
Correction Method: Proza (Phi-Rho-Z)
Acc.Voltage: 20.0 kV Take Off Angle: 29.7 deg.

Quantitative Results for: D68OR-02(1)

Element Line	Net Counts	Net Counts Error		Weight %	Weight % Error	Atom %	Atom % Error	Formula	Compnd %	# Cations
O K	200	+/-	18	16.74	+/- 1.51	29.88	+/- 2.69	O	16.74	---
Na K	1289	+/-	47	10.18	+/- 0.37	12.65	+/- 0.46	Na	10.18	10.155
Al K	148	+/-	34	0.59	+/- 0.14	0.63	+/- 0.14	Al	0.59	0.502
Si K	554	+/-	68	1.81	+/- 0.22	1.84	+/- 0.23	Si	1.81	1.478
S K	1111	+/-	63	3.24	+/- 0.18	2.89	+/- 0.16	S	3.24	2.322
Cl K	11380	+/-	180	39.34	+/- 0.62	31.70	+/- 0.50	Cl	39.34	25.455
K K	4674	+/-	139	21.77	+/- 0.65	15.91	+/- 0.47	K	21.77	12.776
Ca K	1192	+/-	109	6.33	+/- 0.58	4.51	+/- 0.41	Ca	6.33	3.621
Total				100.00		100.00			100.00	56.308

OR 3

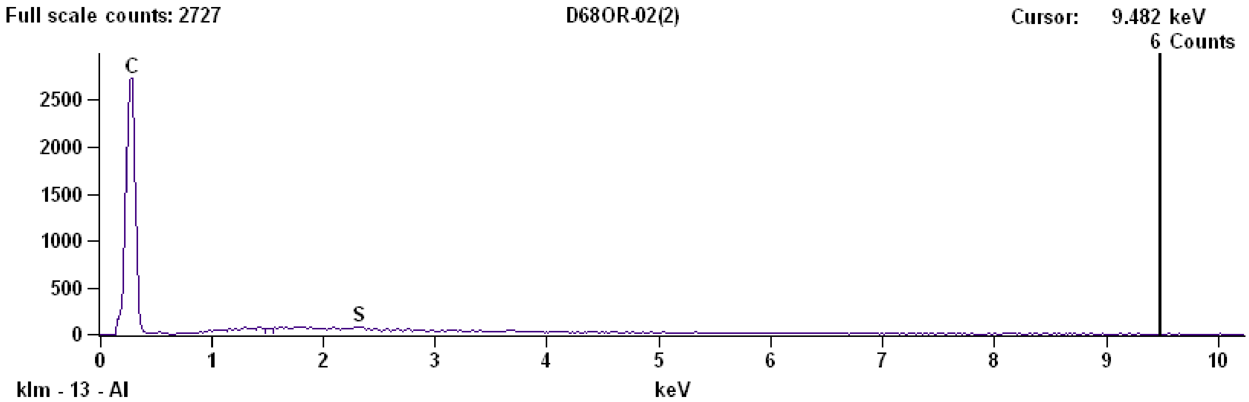

Live Time: 100.0 sec.

Fri Jan 24 13:04:32 2020
Filter Fit Chi Squared:4.690
Errors: +/- 1 Sigma
Correction Method: Proza (Phi-Rho-Z)
Acc.Voltage: 20.0 kV Take Off Angle: 29.7 deg.

Quantitative Results for: D68OR-02(2)

Element Line	*Net Counts*	*Net Counts Error*	*Weight %*	*Weight % Error*	*Atom %*	*Atom % Error*	*Formula*	*Compnd %*	*# Cations*
S K	295	+/- 47	100.00	+/-15.93	100.00	+/-15.93	S	100.00	---
Total			100.00		100.00			100.00	0.000

OR 4

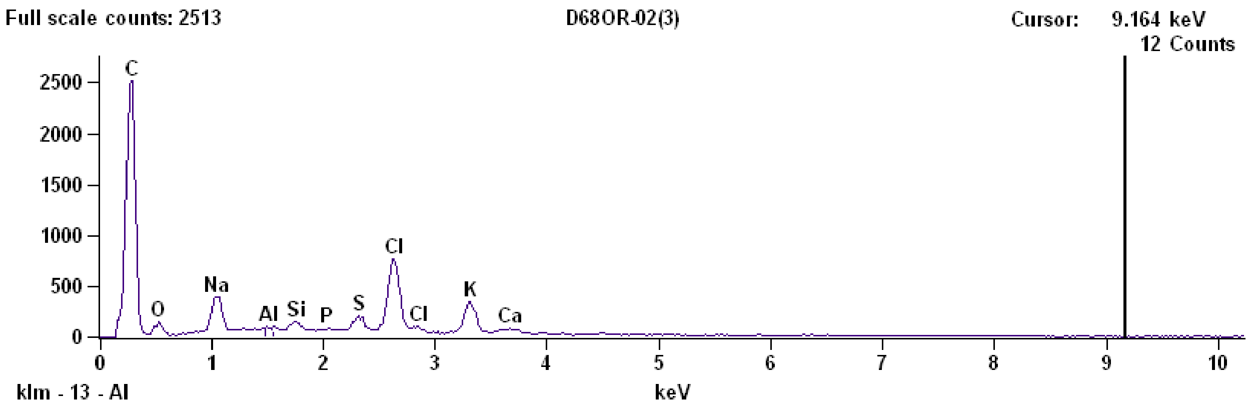

Live Time: 100.0 sec.

Fri Jan 24 13:06:39 2020
Filter Fit Chi Squared:3.934
Errors: +/- 1 Sigma
Correction Method: Proza (Phi-Rho-Z)
Acc.Voltage: 20.0 kV Take Off Angle: 29.7 deg.

Quantitative Results for: D68OR-02(3)

Element Line	Net Counts	Net Counts Error		Weight %	Weight % Error	Atom %	Atom % Error	Formula	Compnd %	# Cations
O K	771	+/-	50	33.28	+/- 2.16	48.85	+/- 3.17	O	33.28	---
Na K	3223	+/-	76	19.72	+/- 0.47	20.15	+/- 0.48	Na	19.72	9.899
Al K	205	+/-	43	0.73	+/- 0.15	0.64	+/- 0.13	Al	0.73	0.314
Si K	794	+/-	78	2.27	+/- 0.22	1.90	+/- 0.19	Si	2.27	0.931
P K	154	+/-	51	0.44	+/- 0.15	0.33	+/- 0.11	P	0.44	0.163
S K	1431	+/-	68	3.57	+/- 0.17	2.62	+/- 0.12	S	3.57	1.286
Cl K	8560	+/-	165	24.63	+/- 0.47	16.32	+/- 0.31	Cl	24.63	8.015
K K	3764	+/-	122	13.09	+/- 0.42	7.86	+/- 0.25	K	13.09	3.863
Ca K	592	+/-	49	2.27	+/- 0.19	1.33	+/- 0.11	Ca	2.27	0.653
Total				100.00		100.00			100.00	25.126

OR 5

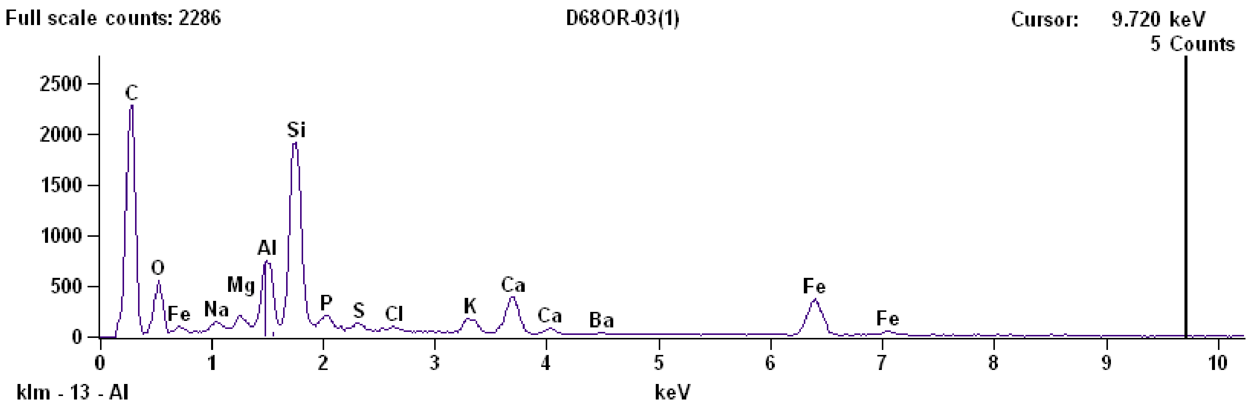

Live Time: 100.0 sec.

Fri Jan 24 13:11:27 2020
Filter Fit Chi Squared:2.279
Errors: +/- 1 Sigma
Correction Method: Proza (Phi-Rho-Z)
Acc.Voltage: 20.0 kV Take Off Angle: 29.7 deg.

Quantitative Results for: D68OR-03(1)

Element Line	Net Counts	Net Counts Error		Weight %	Weight % Error	Atom %	Atom % Error	Formula	Compnd %	# Cations
O K	3550	+/-	93	36.61	+/- 0.96	54.81	+/- 1.44	O	36.61	---
Na K	744	+/-	69	2.17	+/- 0.20	2.27	+/- 0.21	Na	2.17	0.992
Mg K	935	+/-	61	1.65	+/- 0.11	1.63	+/- 0.11	Mg	1.65	0.714
Al K	6778	+/-	151	8.49	+/- 0.19	7.54	+/- 0.17	Al	8.49	3.302
Si K	19826	+/-	221	22.56	+/- 0.25	19.24	+/- 0.21	Si	22.56	8.425
P K	1334	+/-	71	1.91	+/- 0.10	1.48	+/- 0.08	P	1.91	0.648
S K	793	+/-	60	0.95	+/- 0.07	0.71	+/- 0.05	S	0.95	0.311
Cl K	425	+/-	51	0.53	+/- 0.06	0.36	+/- 0.04	Cl	0.53	0.156
K K	1902	+/-	121	2.25	+/- 0.14	1.38	+/- 0.09	K	2.25	0.605
Ca K	4878	+/-	143	6.05	+/- 0.18	3.62	+/- 0.11	Ca	6.05	1.583
Fe K	6068	+/-	157	15.87	+/- 0.41	6.81	+/- 0.18	Fe	15.87	2.981
Ba L	370	+/-	65	0.93	+/- 0.16	0.16	+/- 0.03	Ba	0.93	0.071
Total				100.00		100.00			100.00	19.787

OR 6

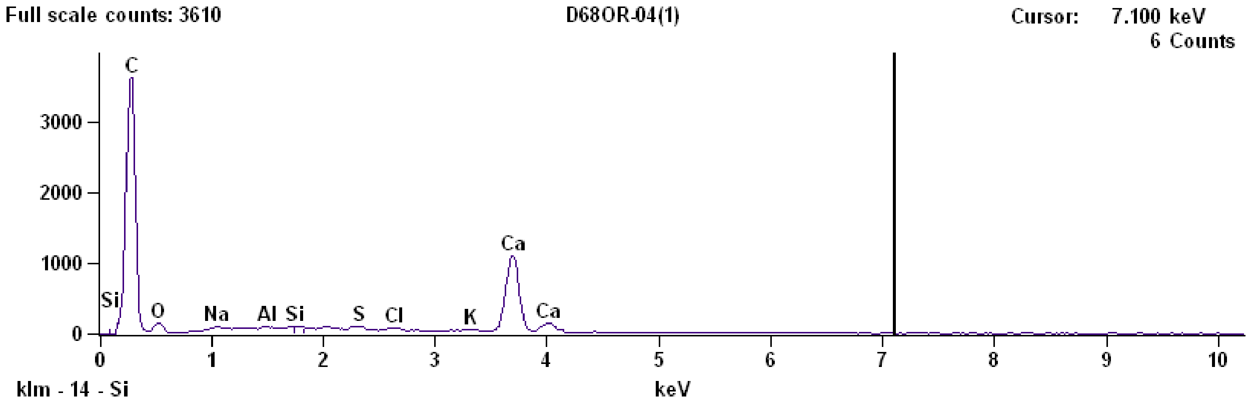

Live Time: 100.0 sec.

Fri Jan 24 13:16:39 2020
Filter Fit Chi Squared:3.681
Errors: +/- 1 Sigma
Correction Method: Proza (Phi-Rho-Z)
Acc.Voltage: 20.0 kV Take Off Angle: 29.7 deg.

Quantitative Results for: D68OR-04(1)

Element Line	*Net Counts*	*Net Counts Error*		*Weight %*	*Weight % Error*	*Atom %*	*Atom % Error*	*Formula*	*Compnd %*	*# Cations*
O K	849	+/-	53	47.30	+/- 2.95	68.01	+/- 4.25	O	47.30	---
Na K	268	+/-	53	2.19	+/- 0.43	2.19	+/- 0.43	Na	2.19	0.773
Al K	203	+/-	42	0.66	+/- 0.14	0.56	+/- 0.12	Al	0.66	0.199
Si K	464	+/-	76	1.21	+/- 0.20	0.99	+/- 0.16	Si	1.21	0.350
S K	707	+/-	54	1.61	+/- 0.12	1.15	+/- 0.09	S	1.61	0.407
Cl K	481	+/-	46	1.21	+/- 0.12	0.78	+/- 0.08	Cl	1.21	0.277
K K	296	+/-	43	0.75	+/- 0.11	0.44	+/- 0.06	K	0.75	0.155
Ca K	15329	+/-	199	45.08	+/- 0.59	25.87	+/- 0.34	Ca	45.08	9.130
Total				100.00		100.00			100.00	11.290

OR 7

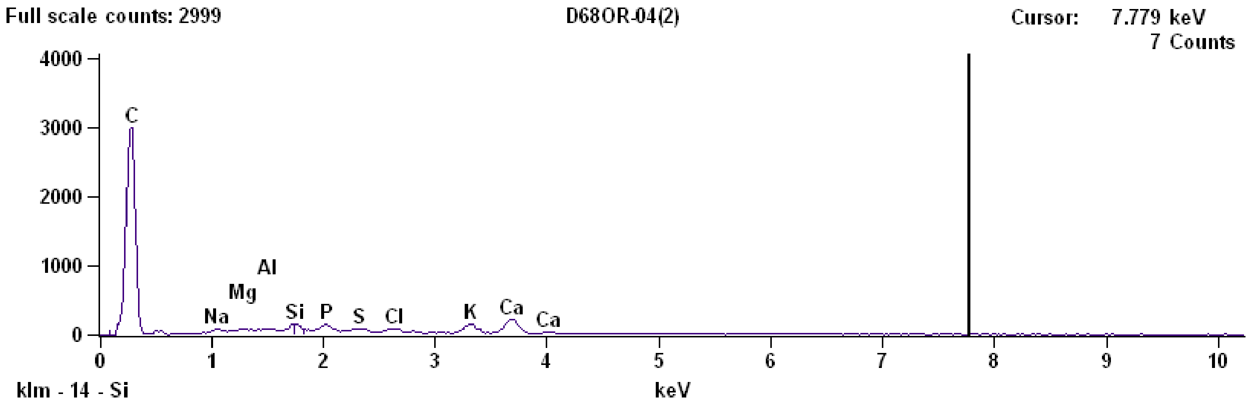

Live Time: 100.0 sec.

Fri Jan 24 13:18:54 2020
Filter Fit Chi Squared:5.001
Errors: +/- 1 Sigma
Correction Method: Proza (Phi-Rho-Z)
Acc.Voltage: 20.0 kV Take Off Angle: 29.7 deg.

Quantitative Results for: D68OR-04(2)

Element Line	Net Counts	Net Counts Error		Weight %	Weight % Error	Atom %	Atom % Error	Formula	Compnd %	# Cations
Na K	291	+/-	50	6.48	+/- 1.11	9.63	+/- 1.65	Na	6.48	---
Mg K	162	+/-	39	2.46	+/- 0.59	3.45	+/- 0.83	Mg	2.46	---
Al K	169	+/-	41	1.89	+/- 0.46	2.39	+/- 0.58	Al	1.89	---
Si K	1091	+/-	79	10.22	+/- 0.74	12.42	+/- 0.90	Si	10.22	---
P K	952	+/-	56	10.15	+/- 0.60	11.19	+/- 0.66	P	10.15	---
S K	546	+/-	52	5.63	+/- 0.54	5.99	+/- 0.57	S	5.63	---
Cl K	574	+/-	47	6.56	+/- 0.54	6.32	+/- 0.52	Cl	6.56	---
K K	1632	+/-	106	18.79	+/- 1.22	16.41	+/- 1.07	K	18.79	---
Ca K	2779	+/-	116	37.81	+/- 1.58	32.20	+/- 1.34	Ca	37.81	---
Total				100.00		100.00			100.00	0.000

BLUE 1

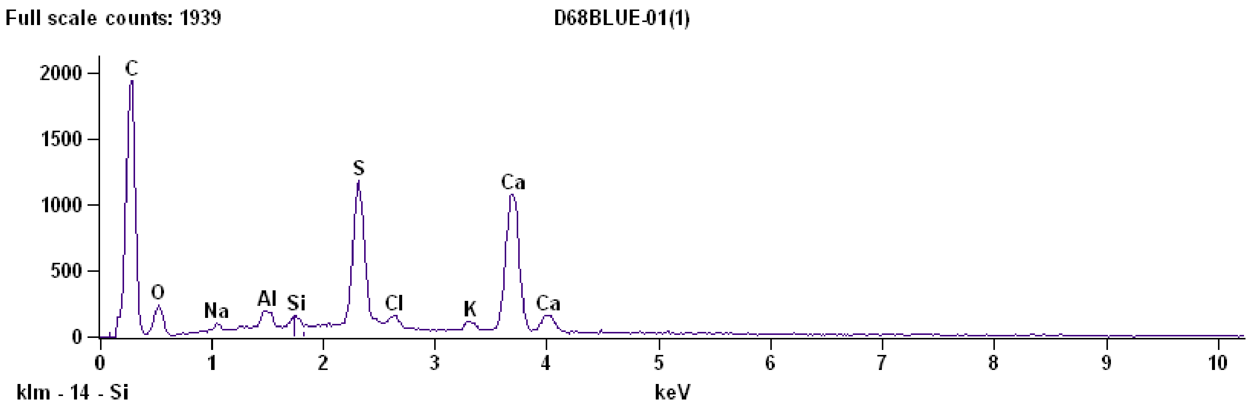

Live Time: 100.0 sec.

Fri Jan 24 13:33:37 2020
Filter Fit Chi Squared:3.592
Errors: +/- 1 Sigma
Correction Method: Proza (Phi-Rho-Z)
Acc.Voltage: 20.0 kV Take Off Angle: 29.6 deg.

Quantitative Results for: D68BLUE-01(1)

Element Line	Net Counts	Net Counts Error		Weight %	Weight % Error	Atom %	Atom % Error	Formula	Compnd %	# Cations
O K	1633	+/-	57	46.19	+/- 1.61	65.38	+/- 2.28	O	46.19	---
Na K	384	+/-	33	1.66	+/- 0.14	1.64	+/- 0.14	Na	1.66	0.601
Al K	1098	+/-	50	1.95	+/- 0.09	1.63	+/- 0.07	Al	1.95	0.600
Si K	649	+/-	51	0.96	+/- 0.08	0.77	+/- 0.06	Si	0.96	0.283
S K	13821	+/-	193	18.24	+/- 0.25	12.89	+/- 0.18	S	18.24	4.731
Cl K	1205	+/-	125	2.11	+/- 0.22	1.35	+/- 0.14	Cl	2.11	0.494
K K	728	+/-	53	1.25	+/- 0.09	0.72	+/- 0.05	K	1.25	0.265
Ca K	14863	+/-	203	27.65	+/- 0.38	15.62	+/- 0.21	Ca	27.65	5.735
Total				100.00		100.00			100.00	12.709

BLUE 2

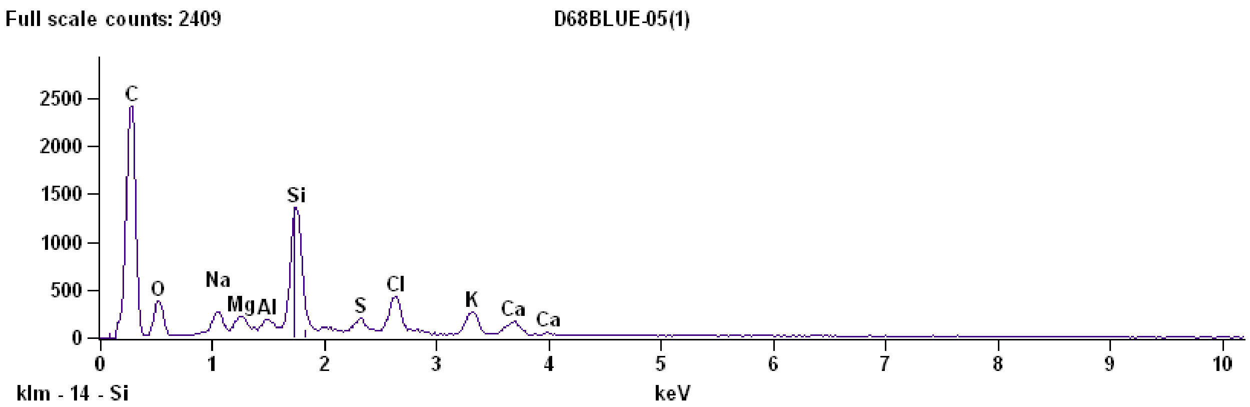

Live Time: 100.0 sec.

Fri Jan 24 13:43:07 2020
Filter Fit   Chi Squared:4.034
Errors: +/- 1 Sigma
Correction Method: Proza (Phi-Rho-Z)
Acc.Voltage: 20.0 kV Take Off Angle: 29.6 deg.

Quantitative Results for: D68BLUE-05(1)

Element Line	Net Counts	Net Counts Error		Weight %	Weight % Error	Atom %	Atom % Error	Formula	Compnd %	# Cations
O K	2775	+/-	72	47.60	+/- 1.23	62.53	+/- 1.62	O	47.60	---
Na K	1985	+/-	76	7.14	+/- 0.27	6.53	+/- 0.25	Na	7.14	2.507
Mg K	1468	+/-	89	3.63	+/- 0.22	3.14	+/- 0.19	Mg	3.63	1.205
Al K	841	+/-	62	1.55	+/- 0.11	1.21	+/- 0.09	Al	1.55	0.463
Si K	13365	+/-	156	20.40	+/- 0.24	15.27	+/- 0.18	Si	20.40	5.861
S K	1615	+/-	119	2.67	+/- 0.20	1.75	+/- 0.13	S	2.67	0.673
Cl K	4717	+/-	140	8.49	+/- 0.25	5.03	+/- 0.15	Cl	8.49	1.932
K K	2796	+/-	125	5.24	+/- 0.23	2.82	+/- 0.13	K	5.24	1.081
Ca K	1640	+/-	116	3.27	+/- 0.23	1.71	+/- 0.12	Ca	3.27	0.658
Total				100.00		100.00			100.00	14.380

BLUE 3

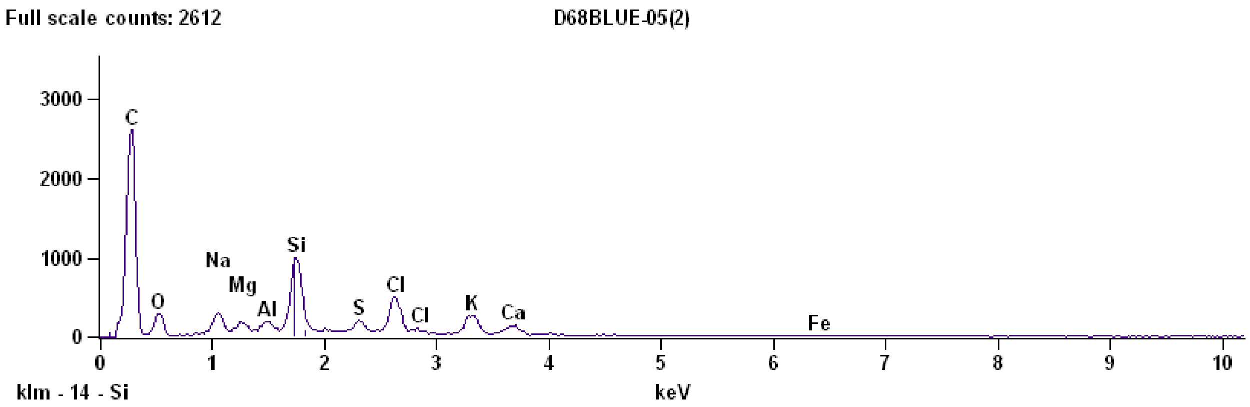

Live Time: 100.0 sec.

Fri Jan 24 13:45:20 2020
Filter Fit   Chi Squared:3.136
Errors: +/- 1 Sigma
Correction Method: Proza (Phi-Rho-Z)
Acc.Voltage: 20.0 kV Take Off Angle: 29.6 deg.

Quantitative Results for: D68BLUE-05(2)

Element Line	Net Counts	Net Counts Error	Weight %	Weight % Error	Atom %	Atom % Error	Formula	Compnd %	# Cations
O K	2073	+/- 65	43.81	+/- 1.37	59.33	+/- 1.86	O	43.81	---
Na K	2348	+/- 75	9.74	+/- 0.31	9.18	+/- 0.29	Na	9.74	3.712
Mg K	811	+/- 56	2.42	+/- 0.17	2.16	+/- 0.15	Mg	2.42	0.873
Al K	869	+/- 57	1.87	+/- 0.12	1.50	+/- 0.10	Al	1.87	0.607
Si K	9646	+/- 138	17.18	+/- 0.25	13.25	+/- 0.19	Si	17.18	5.361
S K	1546	+/- 68	2.86	+/- 0.13	1.93	+/- 0.09	S	2.86	0.782
Cl K	5396	+/- 145	10.93	+/- 0.29	6.68	+/- 0.18	Cl	10.93	2.702
K K	3157	+/- 127	6.81	+/- 0.27	3.77	+/- 0.15	K	6.81	1.526
Ca K	1391	+/- 112	3.21	+/- 0.26	1.74	+/- 0.14	Ca	3.21	0.702
Fe K	251	+/- 40	1.17	+/- 0.19	0.46	+/- 0.07	Fe	1.17	0.184
Total			100.00		100.00			100.00	16.449

Project: Michalik19

BLUE 4

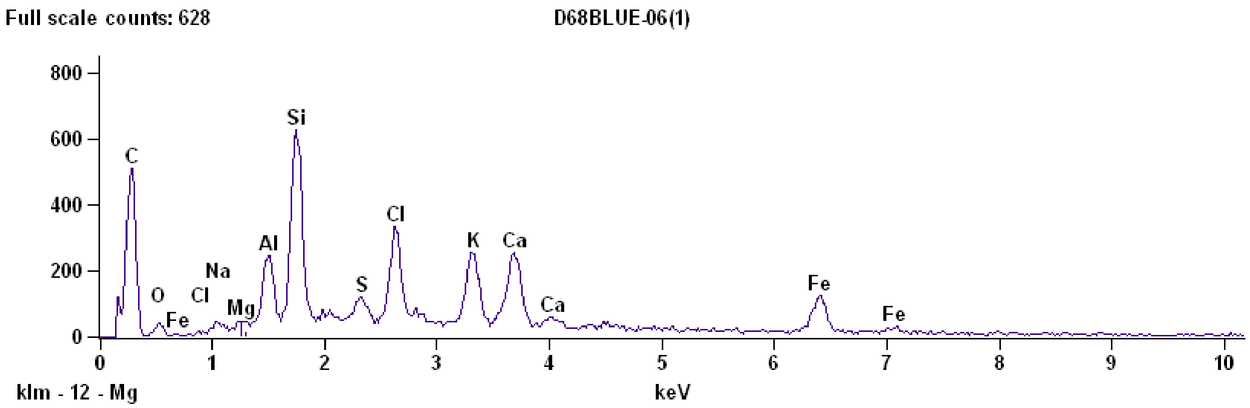

Live Time: 100.0 sec.

Fri Jan 24 13:51:05 2020
Filter Fit Chi Squared:3.035
Errors: +/- 1 Sigma
Correction Method: Proza (Phi-Rho-Z)
Acc.Voltage: 20.0 kV Take Off Angle: 29.3 deg.

Quantitative Results for: D68BLUE-06(1)

Element Line	Net Counts	Net Counts Error		Weight %	Weight % Error	Atom %	Atom % Error	Formula	Compnd %	# Cations
O K	225	+/-	25	14.58	+/- 1.62	26.99	+/- 3.00	O	14.58	---
Na K	174	+/-	21	1.52	+/- 0.18	1.96	+/- 0.24	Na	1.52	1.740
Mg K	137	+/-	33	0.72	+/- 0.17	0.88	+/- 0.21	Mg	0.72	0.780
Al K	2063	+/-	85	7.67	+/- 0.32	8.42	+/- 0.35	Al	7.67	7.488
Si K	6061	+/-	121	20.63	+/- 0.41	21.76	+/- 0.43	Si	20.63	19.349
S K	1006	+/-	99	3.57	+/- 0.35	3.30	+/- 0.32	S	3.57	2.930
Cl K	3322	+/-	121	12.77	+/- 0.47	10.67	+/- 0.39	Cl	12.77	9.490
K K	2883	+/-	125	11.59	+/- 0.50	8.78	+/- 0.38	K	11.59	7.807
Ca K	3192	+/-	128	14.10	+/- 0.57	10.42	+/- 0.42	Ca	14.10	9.265
Fe K	1481	+/-	107	12.87	+/- 0.93	6.83	+/- 0.49	Fe	12.87	6.070
Total				100.00		100.00			100.00	64.919

BLUE 5

Project: Michalik19

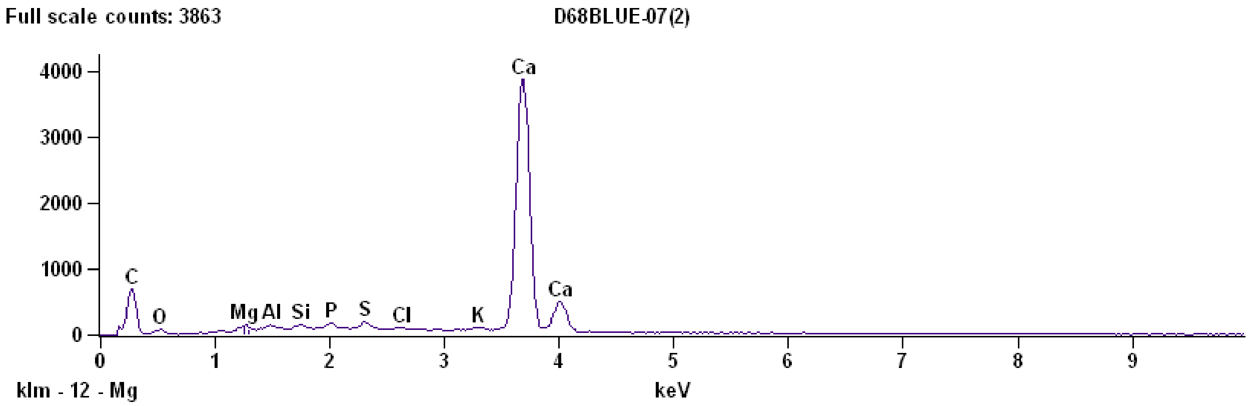

Live Time: 100.0 sec.

Fri Jan 24 13:58:32 2020
Filter Fit Chi Squared:5.013
Errors: +/- 1 Sigma
Correction Method: Proza (Phi-Rho-Z)
Acc.Voltage: 20.0 kV Take Off Angle: 29.8 deg.

Quantitative Results for: D68BLUE-07(2)

Element Line	*Net Counts*		*Net Counts Error*	*Weight %*	*Weight % Error*	*Atom %*	*Atom % Error*	*Formula*	*Compnd %*	*# Cations*
O K	440	+/-	24	18.10	+/- 0.99	34.82	+/- 1.90	O	18.10	---
Mg K	713	+/-	44	1.57	+/- 0.10	1.98	+/- 0.12	Mg	1.57	1.368
Al K	887	+/-	90	1.37	+/- 0.14	1.56	+/- 0.16	Al	1.37	1.078
Si K	802	+/-	56	1.01	+/- 0.07	1.10	+/- 0.08	Si	1.01	0.760
P K	1026	+/-	65	1.27	+/- 0.08	1.26	+/- 0.08	P	1.27	0.871
S K	1559	+/-	123	1.72	+/- 0.14	1.65	+/- 0.13	S	1.72	1.138
Cl K	368	+/-	57	0.44	+/- 0.07	0.39	+/- 0.06	Cl	0.44	0.266
K K	362	+/-	58	0.42	+/- 0.07	0.33	+/- 0.05	K	0.42	0.228
Ca K	52317	+/-	376	74.10	+/- 0.53	56.90	+/- 0.41	Ca	74.10	39.219
Total				100.00		100.00			100.00	44.927

BLUE 6

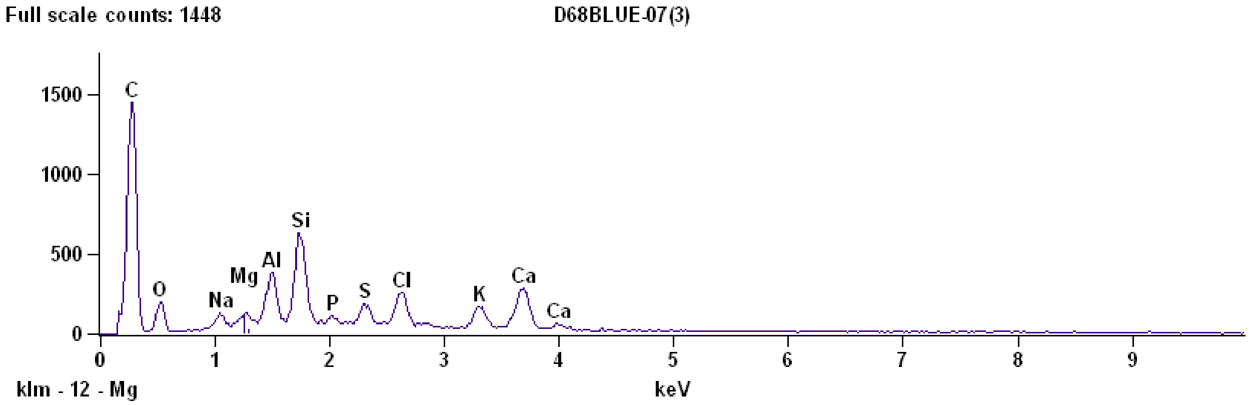

Live Time: 100.0 sec.

Fri Jan 24 14:00:44 2020
Filter Fit Chi Squared:3.956
Errors: +/- 1 Sigma
Correction Method: Proza (Phi-Rho-Z)
Acc.Voltage: 20.0 kV Take Off Angle: 29.8 deg.

Quantitative Results for: D68BLUE-07(3)

Element Line	Net Counts	Net Counts Error		Weight %	Weight % Error	Atom %	Atom % Error	Formula	Compnd %	# Cations
O K	1212	+/-	50	42.51	+/- 1.75	58.72	+/- 2.42	O	42.51	---
Na K	671	+/-	56	4.01	+/- 0.33	3.86	+/- 0.32	Na	4.01	1.576
Mg K	633	+/-	77	2.44	+/- 0.30	2.22	+/- 0.27	Mg	2.44	0.908
Al K	2844	+/-	114	8.10	+/- 0.32	6.63	+/- 0.27	Al	8.10	2.712
Si K	5731	+/-	133	15.15	+/- 0.35	11.92	+/- 0.28	Si	15.15	4.874
P K	331	+/-	55	1.04	+/- 0.17	0.74	+/- 0.12	P	1.04	0.303
S K	1445	+/-	63	3.87	+/- 0.17	2.67	+/- 0.12	S	3.87	1.092
Cl K	2605	+/-	117	7.69	+/- 0.35	4.79	+/- 0.22	Cl	7.69	1.959
K K	1652	+/-	57	5.02	+/- 0.17	2.84	+/- 0.10	K	5.02	1.159
Ca K	3121	+/-	122	10.16	+/- 0.40	5.60	+/- 0.22	Ca	10.16	2.291
Total				100.00		100.00			100.00	16.873

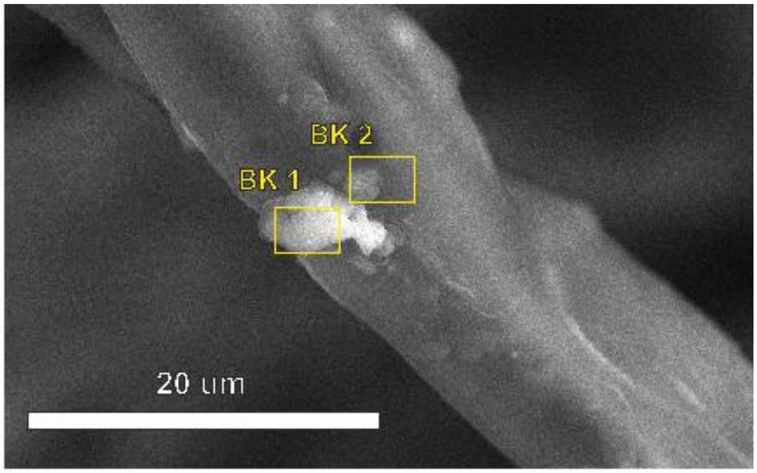
BK 2
BK 1
20 um

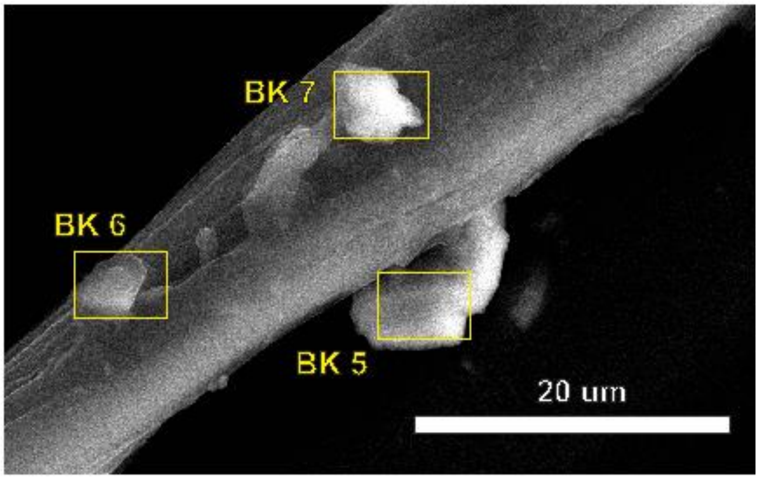
BK 7
BK 6
BK 5
20 um

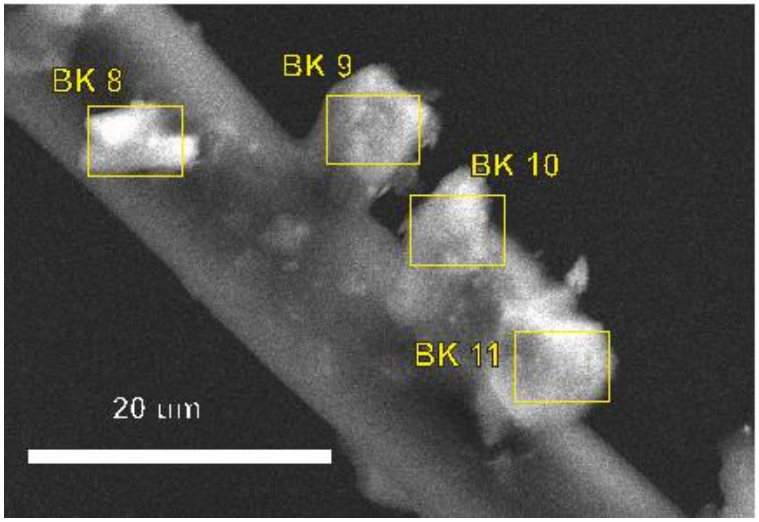
BK 8
BK 9
BK 10
BK 11
20 um

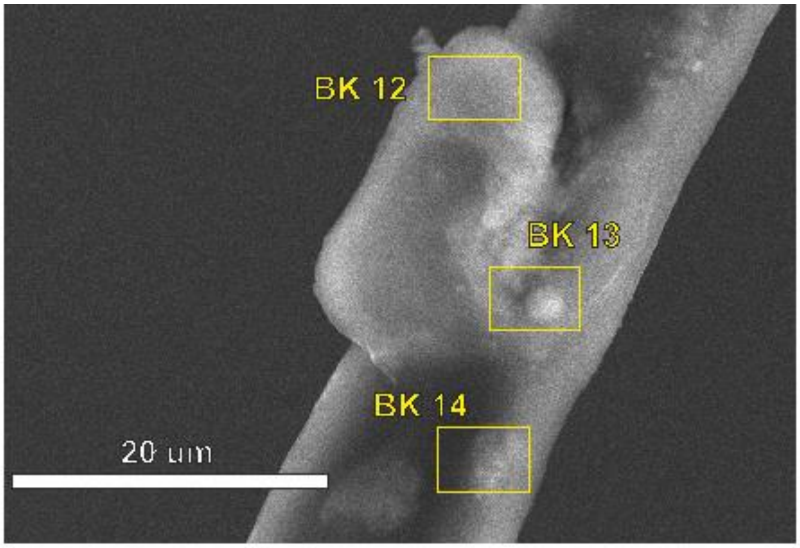
BK 12
BK 13
BK 14
20 um

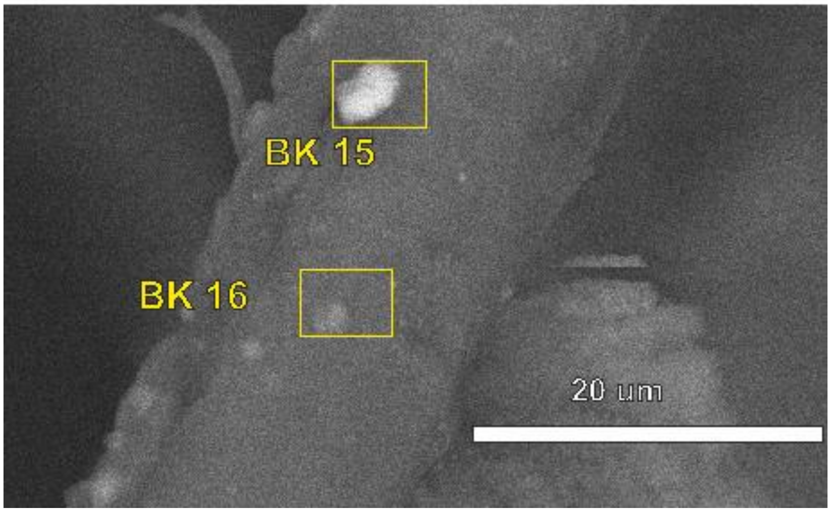
BK 15
BK 16
20 um

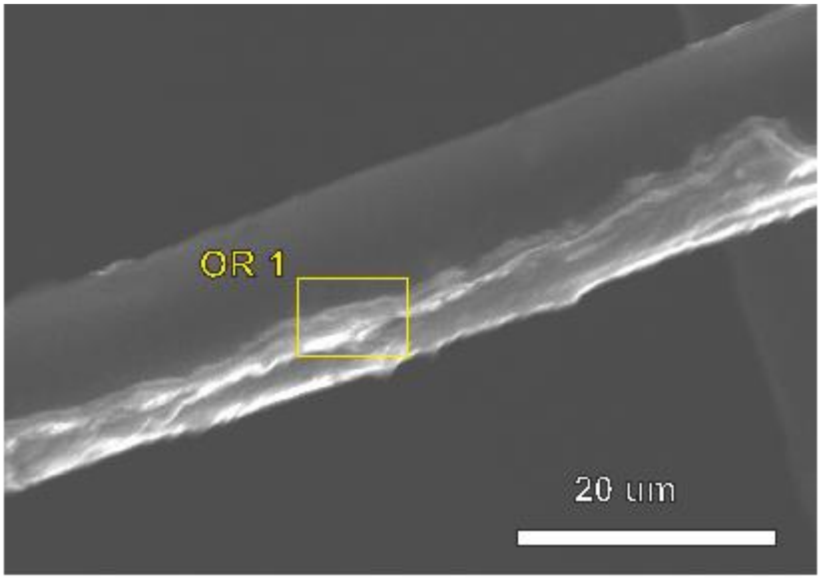
OR 1
20 um

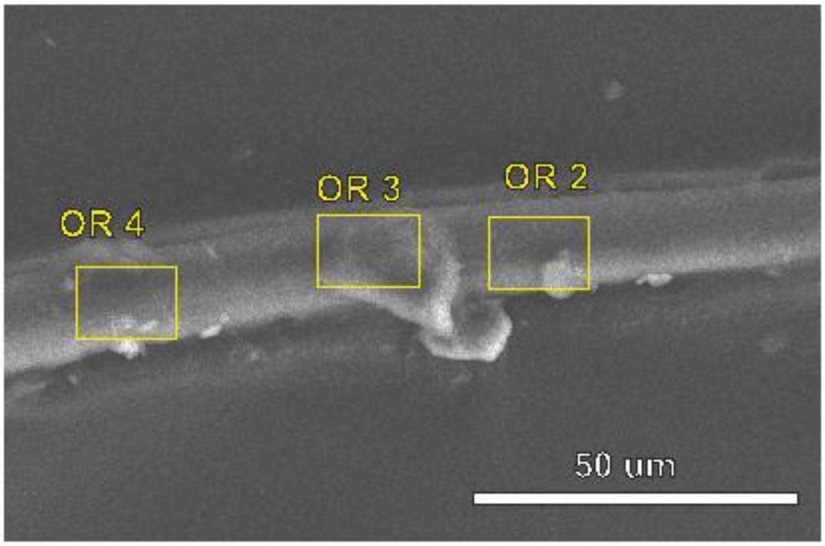
OR 4
OR 3
OR 2
50 um

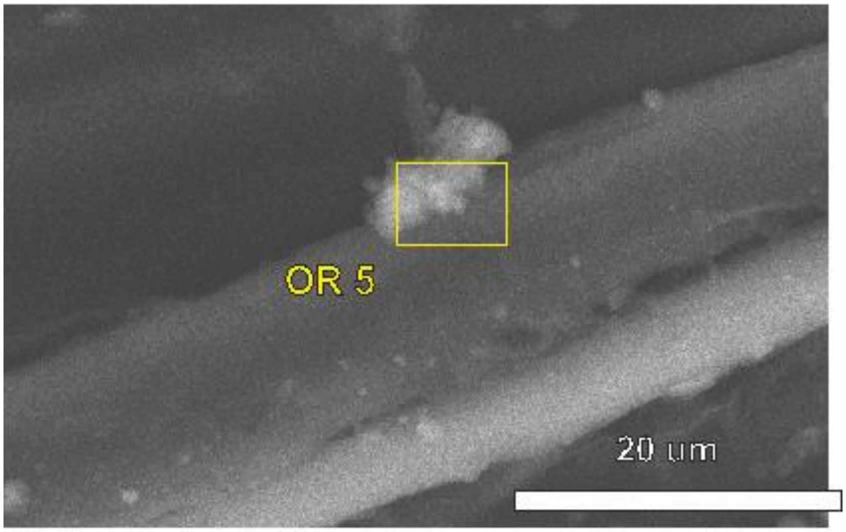
OR 5
20 um

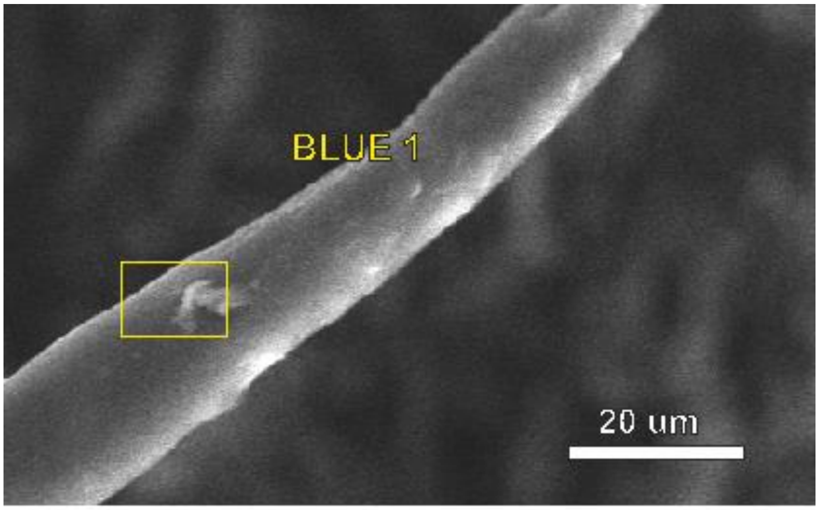
BLUE 1
20 um

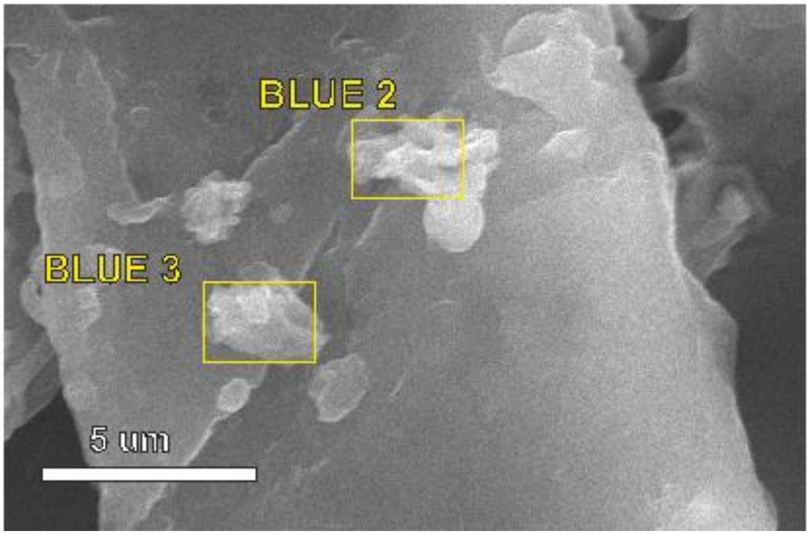
BLUE 2
BLUE 3
5 um

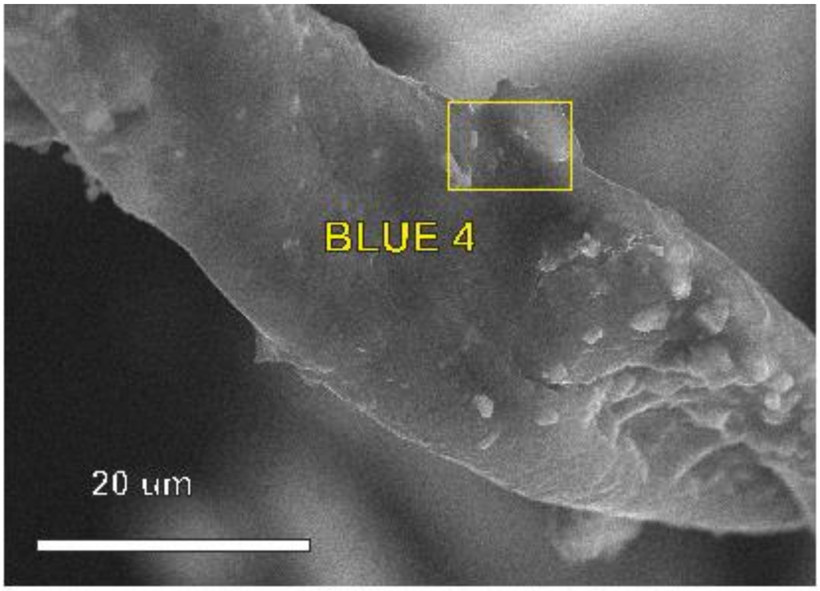
BLUE 4
20 um

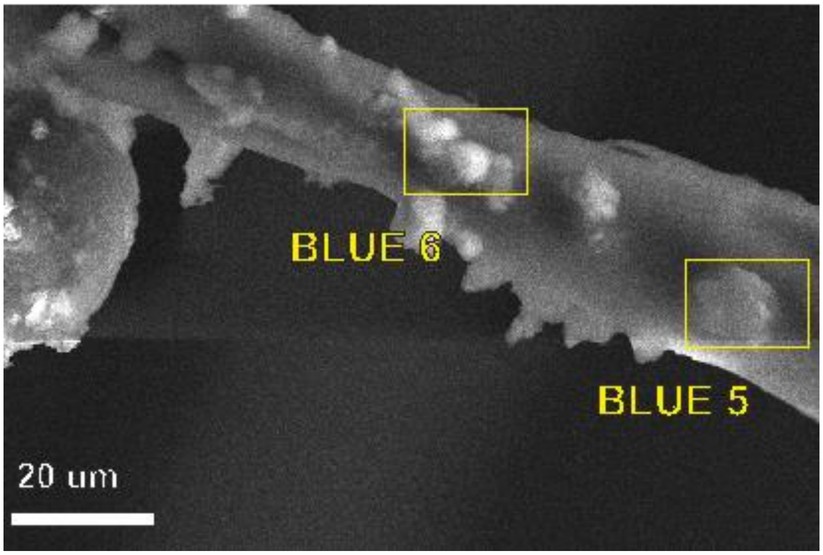
BLUE 6
BLUE 5
20 um
